# Scientific evidence reveals conjoined etiology in spinal deformation and the need for etiology based treatment. Insight in a trail

**DOI:** 10.1186/1748-7161-9-S1-P16

**Published:** 2014-12-04

**Authors:** Piet Van Loon, Andre Grotenhuis

**Affiliations:** 1 Gelre Ziekenhuis, Apeldoorn, Netherlands; 2 Neurosurgery Radboud Medical Centre, Nijmegen, Netherlands

## Introduction

Effects of discongruent growth on the spine and central cord morphology gained attention in Orthopedics. If growth is dependent on exogenic factors and neuromuscular tension, therapy must focus on reversal of this. An extensive search on clues in existing literature was done. Goal was to formulate consequences for treatment strategies.

## Characteristics of earlier research

No doubt European orthopedic science proved that deformities are load dependent changes. Jansen researched sources of rotational forces in the human body produced by the asymmetric diaphragm. Modern research was mainly morphological in nature based on the AP X-ray.

## Opening black box and the holistic concept of Milan Roth

In experiments on the “Nervous Skeleton” and its way of growth Roth offered science on neurovertebral and neuro-osseous growth relations ,with tensionforces. Recent studies with MRI in idiopathic scoliosis confirm much of this11. If there is increased tension in the CNS, there must be increased contraction in muscles too, if their energy is not used to deform the soft connecting parts (Volkmann Hueter principle) or the bones of the spine (Wolff’s Law).12

## Correction by reversing etiologic factors

Own study on forced lordosis we proved two issues towards durable correction: existence of a thoracolumbar kyphosis in scoliosis, confirmed by Ni in Spine and the possibility to correct double curved scoliosis by applying a symmetrical lordotic (and thus extending)force at the TL joint14. By that the erecting muscles are forced back to the midline.

To preserve instantaneous derotation by lordotic forces into real corrective growth faces challenges. By forcing the erecting muscles in normal tracks and create a long lordosis, this position was seen highly unstable but proved to work. In this we oppose Dickson’s axiom not to facilitate lordosis in a deformed spine.

A developed and used lordotic brace applies a complete controlled lordosis. An extra strain on the erector trunci is given through pads giving stability. So normal growth forces of the spine erecting muscles are brought back in their original tracts. TLI braces only prevent flexion, the most prevailing “posture” in modern life of children.

## Result

In a first series were published in Scoliosis. Children can assess their own progress in achieving a better clinical posture and function.

